# Insights into particle dispersion and damage mechanisms in functionally graded metal matrix composites with random microstructure-based finite element model

**DOI:** 10.1038/s41598-024-70247-3

**Published:** 2024-09-06

**Authors:** M. E. Naguib, S. I. Gad, M. Megahed, M. A. Agwa

**Affiliations:** Department of Mechanical Design and Production Engineering, Faculty of Engineering, Zagazig University, P.O. Box 44519, Zagazig, Egypt

**Keywords:** Random microstructure-based model, Functionally graded metal matrix composites, Finite element method, Damage behavior, Spherical indentation, Aerospace engineering, Mechanical engineering, Structural materials, Computational science, Software

## Abstract

This study investigates the impact of $\mathrm {Al_2O_3}$ particle volume fraction and distribution on the deformation and damage of particle-reinforced metal matrix composites, particularly in the context of functionally graded metal matrix composites. In this study, a two-dimensional nonlinear random microstructure-based finite element modeling approach implemented in ABAQUS/Explicit with a Python-generated script to analyze the deformation and damage mechanisms in $\mathrm{AA6061\mbox{-}T6/Al_2O_{3}}$ composites. The plastic deformation and ductile cracking of the matrix are captured using the Gurson–Tvergaard–Needleman model, whereas particle fracture is modelled using the Johnson–Holmquist II model. Matrix-particle interface decohesion is simulated using the surface-based cohesive zone method. The findings reveal that functionally graded metal matrix composites exhibit higher hardness values ($\textrm{HRB}$) than traditional metal matrix composites. The results highlight the importance of functionally graded metal matrix composites. Functionally graded metal matrix composites with a Gaussian distribution and a particle volume fraction of 10% achieve $\textrm{HRB}$ values comparable to particle-reinforced metal matrix composites with a particle volume fraction of 20%, with only a 2% difference in $\textrm{HRB}$. Thus, $\textrm{HRB}$ can be improved significantly by employing a low particle volume fraction and incorporating a Gaussian distribution across the material thickness. Furthermore, functionally graded metal matrix composites with a Gaussian distribution exhibit higher $\textrm{HRB}$ values and better agreement with experimental distribution functions when compared to those with a power-law distribution.

## Introduction

Composite structures have the advantage of exhibiting different material properties at different points, offering the potential to enhance their mechanical behavior^1^. Functionally graded materials ($\textrm{FGMs}$) are a class of advanced structures that possess non-uniform material properties throughout the spatial domain of a material. The advancement of composite materials with graded properties has led to a revolution in the fabrication of engineered components. This is particularly true in industries such as electronics, automobiles, aviation, and biomedicine, where conventional metallic or ceramic matrix composites cannot meet design requirements^2^.

Functionally graded structures can be observed in nature, such as in the bio-tissues of animals and plants. Bones and dental crowns are excellent examples of functionally graded structures because they require a wear-resistant surface combined with a ductile core to withstand high contact and dynamic fatigue loading^3,4^. Although $\textrm{FGMs}$ were initially designed for heat-resistant materials, they have been increasingly employed to control the deformation, pressure, wear, corrosion, and stress concentration by enabling smooth transitional gradients across all dimensions of a product^5^.

Functionally graded metal matrix composites ($\textrm{FGMMCs}$) belong to the category of metal matrix composites ($\textrm{MMCs}$) and are characterized by a continuous variation in the volume fraction ($\mathrm {VF_r}$) of the reinforcement along a specific direction of the matrix alloy^6^. $\textrm{FGMMCs}$ offer a gradual or continuous transition in engineering properties at the macroscopic scale, allowing for the combination of desirable properties without the presence of mechanically weak interfaces, which is a limitation often encountered in surface coating techniques^7^.

**Figure 1:**
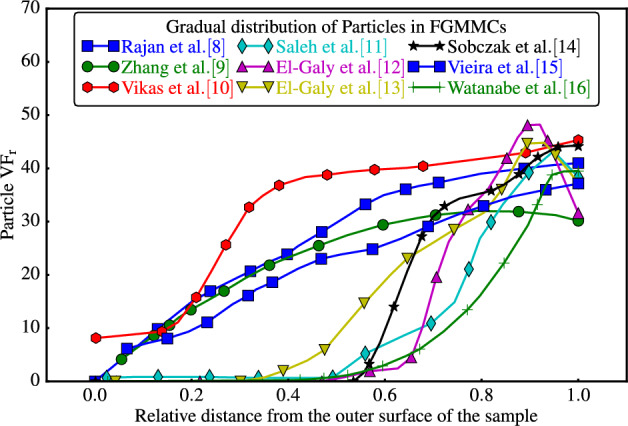
Graded distribution of reinforcing phase in matrix from outer to inner surface based on experimental studies.

A literature review revealed the existence of multiple particle distribution curves within $\textrm{FGMMCs}$; examples are:^8–16^, as depicted in Fig. 1. These empirical curves serve as a valuable resource for theoretical investigations, enabling the development of a controllable function that accurately represents particle distribution within the composite material. Utilizing such a controllable function facilitates a systematic approach for manipulating and analyzing the particle distribution. By fitting the experimental distribution curve to an approximated function, it is possible to obtain a mathematical model that captures the essential characteristics of particle distribution in $\textrm{FGMMCs}$. This model can be integrated into theoretical studies to enable a deeper understanding of the behavior of $\textrm{FGMMCs}$, which can serve as a powerful tool for predicting and optimizing the performance of $\textrm{FGMMCs}$ in practical applications.

To assess the mechanical properties of $\textrm{FGMs}$, the indentation method is often favored owing to its simplicity and minimal specimen preparation requirements. This technique can be easily implemented multiple times on both both small-scale materials and miniaturized structures using a single specimen and a suitable load and indenter tip geometry. The use of numerical modeling to simulate the indentation process of $\textrm{FGMMCs}$ can provide insight into the deformation mechanisms, stress, and strain fields within the metal matrix surrounding the reinforcement particles, as well as the interaction between the particles and the matrix^17^.

Reference^18^ conducted a study wherein graded Al7075/SiC composites were fabricated using a centrifugal casting technique with the aim of enhancing the mechanical properties and wear resistance for automotive purposes. The fabrication process involved utilizing two distinct weight fractions of particles (6.5% and 9.5%), while maintaining a constant mold rotational speed of 1300 rpm. The findings of the study indicated an improvement in the mechanical properties and wear resistance of the composites, especially in the outer region, with an increase in the weight fraction of particles owing to the influence of centrifugal forces.

In the study conducted by^19^, the distribution of SiC particles in aluminum-based $\textrm{FGMMCs}$ used in brake rotor discs was investigated. The average particle size used in this study was 23 ${\upmu }\hbox {m}$. The results demonstrated that the maximum hardness achieved after heat treatment of the Al(356)-SiC and Al(2124)-SiC FGMMCs at the outer periphery was 155 BHN and 145 BHN, respectively. Reference^20^ conducted a study on Al-SiC $\textrm{FGMMCs}$, where they varied the volume percentage of reinforcement. They found that, as the percentage of reinforcement increased, the density decreased. However, they observed that the hardness and wear resistance increased from the core to the cast surface. At low sliding speeds, they noted that microcracking and abrasive wear were dominant factors.

Reference^21^ conducted an investigation into the effects of particle segregation ratio and particle distribution on the $\mathrm {VF_r}$ and particle size ($\textrm{PS}$) of reinforcement in aluminum based $\textrm{FGMMCs}$ with in-situ primary $\mathrm {Si/Mg_2Si}$ particles. The study involved varying parameters, such as the mold temperature and high pouring temperature. The fabricated $\textrm{FGMMCs}$ showed a graded dispersion of primary Si and $\mathrm {Mg_2Si}$ particles in the inner region, with diameters ranging from 70 to 30 ${\upmu }\hbox {m}$ and 30 to 18 ${\upmu }\hbox {m}$, respectively. Additionally, the volume fractions of $\mathrm {Mg_2Si}$ and primary Si exhibited a gradient distribution ranging from $\mathrm {VF_r}$ of 14.8 to 27.7%. The highest hardness values were observed in the middle portion of the inner layer of the fabricated $\textrm{FGMMCs}$, ranging from Rockwell hardness ($\textrm{HRB}$) 72.0 to 75.0. Conversely, the outer layer exhibited the lowest hardness value of approximately $\textrm{HRB}$ 56.0 63.0.

Reference^22^ enhanced the resistance to contact damage in a graded material system comprising silicon nitride ($\mathrm {Si_3N_4}$) ceramic (higher modulus) and oxynitride glass (lower modulus) by employing a unidirectional gradient of the elastic modulus from the contact surface to the interior. They achieved a 30% reduction in the maximum tensile stresses outside the Hertzian contact circle compared with the monolithic material ($\mathrm {Si_3N_4}$) of the graded system.

Reference^23^ investigated the effectiveness of two-scale modeling for analyzing the mechanical properties of $\textrm{FGMs}$ and evaluated aluminum as the supporting matrix and silicon carbide as particle inclusions. The mechanical properties of the material were derived at the macroscale level using a full model, and at the microscale level using a representative volume element in two-scale modeling. The results demonstrated the usefulness and reliability of the two-scale model in reducing the numerical computational time. As the $\mathrm {VF_r}$ of the inclusion increases, the deviation in the stress values decreases, providing evidence supporting the theory that $\textrm{FGMs}$ offer the advantage of smoother stress distributions.

Reference^17^ conducted a study on the indentation behavior of $\textrm{FGMMCs}$, revealing that the number of layers, compositional gradient exponent, and random particle dispersion have a significant impact on the properties of material. Increasing the number of layers resulted in noticeable increases in indentation depths, whereas increasing the compositional gradient exponent led to higher mean residual stresses. Conversely, at a specific layer number, an increase in the compositional gradient exponent decreased the mean residual stresses and strains owing to an increase in the ceramic $\mathrm {VF_r}$. Random particle dispersion influences the central indentation depth and deformed surface profiles, resulting in non-uniform levels and distributions of residual stress and strain.

To the best of our knowledge, no computational $\textrm{FE}$ model has been developed thus far to quantitatively analyze the evolution of multiple damage mechanisms during the indentation of $\textrm{FGMMCs}$. The development of such a model is crucial for predicting material behavior under various conditions by systematically analyzing failure mechanisms and simulating responses to stress and strain over time. These models are essential for optimizing the material performance because they enable the tailoring of microstructures to enhance durability and resilience, thus reducing the need for extensive physical testing. They manage complex geometries and loading conditions, providing a virtual testing environment that identifies potential failure points and predicts the real-world performance. Integrating finite element damage models into research and development not only improves cost efficiency and innovation but also deepens material understanding and facilitates advancements in material engineering across various industries. The objective of this study is to address the gap in stress-state-dependent quantitative damage growth analysis in $\textrm{FGMMCs}$ by investigating the indentation of $\mathrm{AA6061\mbox{-}T6/Al_2O_3}$ $\textrm{FGMMCs}$. To achieve this, a $\textrm{2D}$ microstructure-based $\textrm{FE}$ model is employed to closely approximate the actual microstructure and consider all three potential failure modes. The hardness assessment procedure outlined in the $\mathrm{ASTM~E18\mbox{-}22}$ standard^24^ is incorporated into finite element analysis ($\textrm{FEA}$). To capture the damage mechanisms in the $\mathrm{AA6061\mbox{-}T6}$ matrix, the Gurson–Tvergaard–Needleman ($\textrm{GTN}$) model is employed. This model represents the behavior of the matrix considering void growth and coalescence. Simultaneously, the Johnson–Holmquist II ($\textrm{JH2}$) model is integrated into the FE model to represent $\mathrm {Al_2O_3}$ particle cracking. The matrix-particle interfacial behavior is further modeled using the cohesive zone method ($\textrm{CZM}$). The analysis is conducted using ABAQUS/Explicit $\textrm{FE}$ software, complemented by Python for scripting and preprocessing^25,26^.

## Problem formulation

**Figure 2:**
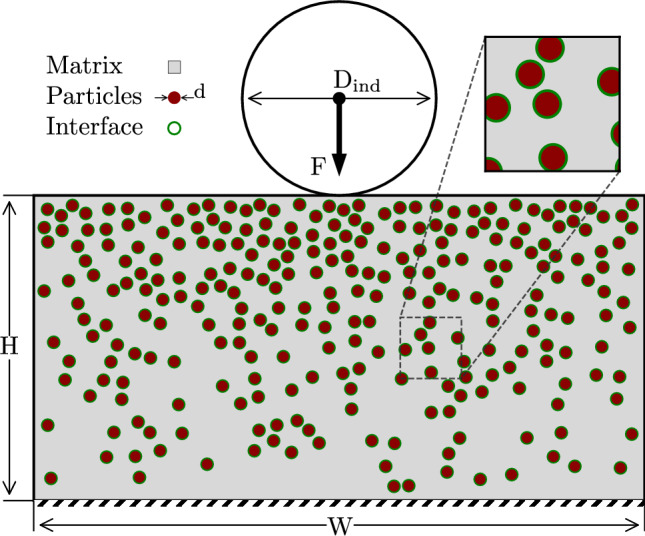
Model configuration of FGMMCs.

Figure 2 depicts the configuration of an $\mathrm{AA6061\mbox{-}T6/Al_2O_3}$ $\textrm{FGMMCs}$ with a spherical indenter. The composite structure is simulated as a rectangle with a width ($\textrm{W}$) of ${5}\,\hbox {mm}$ and height ($\textrm{H}$) of ${2.5}\,\hbox {mm}$. In accordance with the ASTM E18-22 standard^24^, the diameter of indenter ($\mathrm {D_{ind}}$) and applied force ($\textrm{F}$) were determined as ${1.558}\,\hbox {mm}$ and 980 N, respectively. The $\textrm{HRB}$ was computed using the following equation

1 $$\begin{aligned} \textrm{HRB}=130-\frac{h}{0.002} \end{aligned}$$

where *h* represents the permanent penetration in millimeters (mm).

### Material constitutive models

This study focuses on investigating a composite material consisting of $\mathrm{AA6061\mbox{-}T6}$ alloy, which is chosen as the base matrix because of its extensive range of engineering and structural applications^7^. The composite is reinforced with $\mathrm {Al_2O_3}$ particulates, which are the most commonly used reinforcement for $\textrm{PRMMCs}$^27^ with volume fractions of 10% and 20% and an average size of ${100}\,{\upmu }\hbox {m}$. In general, composite materials can fail because of one of the three major failure modes. These modes include plastic deformation and ductile cracking of the matrix, fracture of the reinforcement particles, and decohesion at the matrix-particle interface. The following subsections provide a detailed description of each failure mode.

#### Plastic deformation and ductile cracking of the matrix

A three-stage mechanism involving void nucleation, growth, and coalescence is responsible for the ductile damage observed in the metals. These voids can be initiated by inclusions or microcracks. Subsequently, these voids undergo enlargement owing to the accumulation of plastic strain. The $\textrm{GTN}$ model^28^ is commonly employed for the theoretical analysis of this phenomenon in porous metals. The $\textrm{GTN}$ model is a modified version of the Gurson model^29^ and its yield function is mathematically expressed as

2 $$\begin{aligned} \varphi (\sigma , f)=\left( \frac{\sigma _q}{\sigma _y}\right) ^2+2 q_1 f^* \cosh \left( \frac{3 q_2 p}{2 \sigma _y}\right) -\left( 1+q_3 f^{* 2}\right) =0, \end{aligned}$$

where the non-dilatational strain energy is denoted by $\varphi$, and constants $q_1$, $q_2$, and $q_3$ are introduced by Tvergaard^30^ to account for void interactions. Von Mises and flow stresses of the intact material are represented by $\sigma _q$ and $\sigma _y$, respectively.

To accurately capture the rapid reduction in stress-carrying capacity resulting from void coalescence, Tvergaard–Needleman^28^ introduced the parameter $f^{*}$, known as the effective porosity. This parameter acts as a modeling tool and is mathematically defined as follows:

3 $$\begin{aligned} f^*= {\left\{ \begin{array}{ll}f &{} f \le f_c \\ f_c+\frac{f_u^*-f_c}{f_F-f_c}\left( f-f_c\right) &{} f_c \le f \le f_F, \\ f_u^* &{} f \ge f_F\end{array}\right. } \end{aligned}$$

The $\textrm{GTN}$ model incorporates several parameters to describe void behavior. The critical void volume fraction ($\textrm{VVF}$) at the beginning of coalescence is denoted by $f_c$ and $f_{u}^{*}=\frac{1}{q_1}$ represents the $\textrm{VVF}$ at which the material loses its stress-bearing capacity. Meanwhile, $f_F$ represents the $\textrm{VVF}$ at which the material experiences complete failure and controls the element deletion process. The increase in the $\textrm{VVF}$ owing to void nucleation and growth is considered. The function describing the effective porosity can be expressed as follows:

4 $$\begin{aligned} \textrm{d} f=\textrm{d} f_{\text{ n }}+\textrm{d} f_{\text {g}}. \end{aligned}$$

The rate of void growth ($\textrm{d} f_{\text {g}}$) can be mathematically represented as a function of plastic volume change when the material is considered plastically incompressible.

5 $$\begin{aligned} \textrm{d} f_{\text{ g }}=(1-f)\textrm{d}\varepsilon _{ii}^{P}, \end{aligned}$$

where the trace of the plastic strain-rate tensor is expressed by $\textrm{d}\varepsilon _{ii}^{P}$. The nucleation of voids is strongly influenced by plastic strain, particularly under hydrostatic tension^31^. This phenomenon can be mathematically represented as follows

6 $$\begin{aligned}&\textrm{d} f_{\text{ n }}=A_n \textrm{d} \bar{\varepsilon }_m^p, \end{aligned}$$

7 $$\begin{aligned}&A_n= {\left\{ \begin{array}{ll}\frac{f_N}{S_N \sqrt{2 \pi }} e^{-0.5\left( \frac{\bar{\varepsilon }^p-\varepsilon _N}{S_N}\right) ^2} &{} \text{ if } p \ge 0, \\ 0 &{} \text{ if } p<0\end{array}\right. } \end{aligned}$$

Variable *p* represents the hydrostatic stress, and $f_N$ represents the volume fraction of nucleated voids. Additionally, $\varepsilon _N$ signifies the mean equivalent plastic strain for void nucleation and $s_N$ represents the standard deviation of the void distribution. The macroscopic plastic work rate can be equivalent to the rate of matrix plastic dissipation, which in turn determines the rate of equivalent plastic strain $\textrm{d}\bar{\varepsilon }^p$ according to the following equation

8 $$\begin{aligned} \textrm{d} \bar{\varepsilon }^p=\frac{\sigma : \textrm{d} \varepsilon ^p}{(1-f) \sigma _y}. \end{aligned}$$

Table 1 presents the elastoplastic and $\textrm{GTN}$ model constants employed for the $\mathrm{AA6061\mbox{-}T6}$ matrix derived from previous studies.

#### Fracture of reinforcement particles

The JH2 model constitutive model was initially employed to simulate the mechanical response of materials exhibiting brittle fractures, particularly ceramic materials. Based on the foundational principles outlined in^32^, the JH2 model incorporates the mechanisms of softening and pressure-dependent strength, material damage, and fracture; it also accounts for significant residual strength after fracture, bulking, and sensitivity to the loading rate. The $\textrm{JH2}$ model characterizes the behavior of materials in their damaged state by considering three distinct states: intact, damaged, and fractured. In this context, the model employs an analytical function to express the normalized equivalent stress in the damaged state. The generic form of this function is as follows

9 $$\begin{aligned} \sigma ^*=\sigma _i^*-D\Big (\sigma _i^*-\sigma _f^*\Big )=\sigma /\sigma _{HEL}, \end{aligned}$$

$\sigma _i^*$, $\sigma _f^*$, $\textrm{D}$, and $\sigma _{HEL}$ are defined as follows: $\sigma _i^*$ denotes the normalized intact equivalent stress; $\sigma _f^*$ represents the normalized fracture stress; $\textrm{D}$ is the damage factor, which varies between 0 and 1.0; and $\sigma _{HEL}$ denotes the equivalent stress at the Hugoniot Elastic Limit ($\textrm{HEL}$). The critical point signifies the net compressive stress, which considers both hydrostatic pressure and deviatoric stress components. This is the point at which a one-dimensional shock wave with uniaxial strain exceeds the elastic limit of the material^33^. Lastly, $\sigma$ denotes the actual equivalent stress.

The normalized intact strength ($\sigma _i^*$) can be determined using the following equation:

10 $$\begin{aligned} \sigma _i^*=A(P^*+T^*)^N\left( 1+C\cdot \ln \dot{\varepsilon }^*\right) . \end{aligned}$$

where *A*,  *C*,  *and* *N* are the material constants. $P^*$ represents the normalized pressure defined as the ratio of the actual hydrostatic pressure (*P*) to the hydrostatic pressure at the $\textrm{HEL}$ ($P_{HEL}$). $T^*$ represents the normalized maximum tensile hydrostatic pressure, which is defined as the ratio of the maximum tensile hydrostatic pressure that the material can withstand (*T*), to $P_{HEL}$. $\dot{\varepsilon }^*$ represents the dimensionless strain rate, defined as the ratio of the actual equivalent strain rate ($\dot{\varepsilon }$) to the reference strain rate ($\dot{\varepsilon }_0 = 1.0\;s^{-1}$).

Similarly, the equation for the normalized fracture strength ($\sigma _f^*$) can be expressed as

11 $$\begin{aligned} \sigma _f^*=B(P^*)^M\Big (1+C\cdot \ln \dot{\varepsilon }^*\Big )\le \textrm{SFMAX}, \end{aligned}$$

where *B*,  *C*,  *and* *M* are the material constants. $\textrm{SFMAX}$ represents the ultimate value of the normalized fracture strength ($\sigma ^*_f$), providing additional flexibility in the definition of the fracture strength.

According to the $\textrm{JH2}$ model, the softening process in brittle materials can be described by Eq. (9), which allows for the gradual softening of the material as the plastic strain increases. The softening process continues until the material is fully damaged ($D_p~=~1$). The expression describing the cumulative damage resulting from fracture is expressed as follows

12 $$\begin{aligned} D_p=\sum \frac{\Delta \varepsilon ^P}{\varepsilon _f^P}=\sum \frac{\Delta \varepsilon ^P}{\left[ d_1(P^*+T^*)^{d_2}\right] }, \end{aligned}$$

where $\Delta \varepsilon ^P$ represents the accumulated plastic strain during the integration cycle. Function $\varepsilon _f^P=f(P)$ represents the plastic strain necessary for fracture under a constant pressure *P*. The parameters $d_1$ and $d_2$ correspond to the damage factors associated with $\varepsilon _f^P$.

When a material reaches a certain threshold of plastic deformation or damage, it enters a failure state described by fluid-like behavior^33^. In this state, the material loses its strength and cannot withstand the stress. Both the hydrostatic pressure and deviatoric stress become zero. The relationship between hydrostatic pressure *P* and volumetric strain $\mu$ is described by a polynomial equation of state ($\textrm{EOS}$), which consists of two distinct stages: an elastic stage and a plastic damage stage. The mathematical expressions for these stages are as follows

13 $$\begin{aligned} {\left\{ \begin{array}{ll} P=K_1\cdot \mu +K_2\cdot \mu ^2+K_3\cdot \mu ^3 &{}{D_p=0}\\ P=K_1\cdot \mu +K_2\cdot \mu ^2+K_3\cdot \mu ^3+\Delta P&{}(0<D_p\le 1)\end{array}\right. }, \end{aligned}$$

Here, $K_1$, $K_2$, and $K_3$ are constants and $\mu$ is defined as the ratio of the current density ($\rho$) to the initial density ($\rho _0$). For tensile pressure ($\mu <0$), Eq. (13) is replaced by $P = K_1 \cdot \mu$. The incremental pressure ($\varDelta P$) is added when the material fractures owing to bulking energy.

The reduction in incremental internal elastic energy is transformed into potential internal energy via an incremental increase in hydrostatic pressure $\varDelta P$. As the fracture progresses, the shear and deviatoric stresses diminish owing to the decrease in the equivalent plastic flow stress $\sigma$. The elastic internal energy related to these shear and deviatoric stresses can be expressed mathematically as

14 $$\begin{aligned} U=\frac{\sigma ^2}{6G}, \end{aligned}$$

where *G* denotes the rigidity modulus. The incremental energy loss is given by

15 $$\begin{aligned} \Delta U=U_{D(t)}-U_{D(t+\Delta t)}, \end{aligned}$$

The quantities $U_{D(t)}$ and $U_{D(t+\Delta t)}$ are determined using Eq. (14). The change in energy, $\varDelta U$, is primarily converted into incremental fracturing energy, $\varDelta F$. An approximate expression for this energy conversion is given by

16 $$\begin{aligned} \Delta U=U_{D(t)}-U_{D(t+\Delta t)}, \end{aligned}$$

where $\beta$ is a fraction satisfying $(0 \le \beta \le 1)$, which represents the extent of energy transformation. The $\textrm{JH2}$ model constants used for the $\mathrm {Al_2O_3}$ particles are summarized in Table 1.

**Table 1:** Material constitutive models parameters utilized in this study.

$\mathrm {AA6061-T6}$ (GTN model) References^30,34,35^	Value	$\mathrm {Al_2O_3}$ particles (JH2 model) References^36,37^	Value
E	70 GPa	A	0.93
$\mathrm {S_y}$	275 MPa	B	0.31
$\mathrm {S_{ult}}$	361 MPa	G	155 GPa
$q_1$	1.5	T	0.6 GPa
$q_2$	1	N	0.6
$q_3$	2.25	HEL	10.5 GPa
$f_0$	0.001	$P_{HEL}$	4.5 GPa
$f_c$	0.02	M	0.6
$f_F$	0.06	C	0.0
$\varepsilon _N$	0.03	$\sigma _{i,max}$	12.2 GPa
$S_N$	0.1	$\sigma _{f,max}$	1.3 GPa
$f_N$	0.0005	$\mathrm {K_1}$	193 GPa
–	–	$\mathrm {K_2}$	0.0 GPa
–	–	$\mathrm {K_3}$	0.0 GPa
–	–	$\varepsilon _{f,min}$	0.0
–	–	$\varepsilon _{f,max}$	1.2
–	–	$d_1$	0.005
–	–	$d_2$	1.0
–	–	FS	0.2
–	–	$\beta$	1.0
–	–	$\rho$	3890 $\mathrm {kg/m^3}$

#### Cohesive zone modeling (CZM) for matrix and particle interfaces

The failure process of an interface surface involves three fundamental components: initiation of damage, progression of damage, and overall debonding resulting from substantial damage. The cohesive zone method ($\textrm{CZM}$) is utilized to describe the potential debonding occurring at the interface between the particles and matrix. This study characterizes the $\textrm{CZM}$ through a bilinear relationship between traction ($\textrm{T}$) and separation ($\Delta$). The initiation of damage is governed by the quadratic stress failure criterion. This criterion postulates that damage is initiated when a quadratic interaction function reaches a unit value^38^. Mathematically, this criterion can be expressed as

17 $$\begin{aligned} \left( \frac{T_n}{T_n^0}\right) ^2+\left( \frac{T_s}{T_s^0}\right) ^2+\left( \frac{T_t}{T_t^0}\right) ^2=1, \end{aligned}$$

where $T_n^0$, $T_s^0$, and $T_t^0$ represent the peak values of the nominal stresses during deformation along specific directions: normal to the interface, the first shear direction, and the second shear direction, respectively.

In the traction-separation model, it is assumed that the scalar damage variable $\textrm{D}$ changes from 0 to 1 as the material undergoes further loading after damage initiation. The damage variable $\textrm{D}$ exerts the following influence on the stress components:

18 $$\begin{aligned}&T_n={\left\{ \begin{array}{ll}(1-D)\sigma _n^*&{}\sigma _n^*\ge 0\\ \sigma _n^*&{}\sigma _n^*<0\end{array}\right. }, \end{aligned}$$

19 $$\begin{aligned}&T_s=(1-D)\sigma _s^*,\end{aligned}$$

20 $$\begin{aligned}&T_t=(1-D)\sigma _t^*, \end{aligned}$$

The stress components $\sigma _n^*$, $\sigma _s^*$, and $\sigma _t^*$ are obtained by utilizing the elastic traction-separation behavior for the current strain before damage initiation. To characterize the progression of damage within the interface under combined normal and shear deformations, it is helpful to introduce an effective displacement $\Delta _m$. The effective displacement is defined as follows

21 $$\begin{aligned} \Delta _m=\sqrt{\Delta _n^2+\Delta _s^2+\Delta _t^2}, \end{aligned}$$

The nominal separations in the normal ($\Delta _n$) and in-plane shear ($\Delta _s$, $\Delta _t$) directions govern the evolution of damage variable $\textrm{D}$ through the following expression

22 $$\begin{aligned} \begin{aligned} D =\left\{ \begin{array}{l l}{{0}}&{}{{\Delta _m^{max}}\le \Delta _{m}^{0}}\\ {{\frac{\Delta _{m}^{f}\left( \Delta _{m}^{{max}}-\Delta _{m}^{0}\right) }{\Delta _{m}^{{max}}\left( \Delta _{m}^{f}-\Delta _{m}^{0}\right) }}}&{}{{\Delta _{m}^{0}<\Delta _{m}^{{max}}<\Delta _{m}^{f},}}\\ {{1}}&{}{{\Delta _{m}^{{max}}>\Delta _{m}^{f}}}\end{array}\right. \\ \end{aligned} \end{aligned}$$

where $\Delta _m^0$ and $\Delta _m^f$ characterize the effective separations at the initiation of damage and complete failure, respectively, and $\Delta _m^{max}$ represents the peak value of the effective displacement attained during the loading history. The fracture energy, $G_c$, which is a measure of the area enclosed by the traction-separation displacement curve, can be obtained as follows

23 $$\begin{aligned} G_c =\frac{1}{2}T_n^0\Delta _m^f. \end{aligned}$$

The CZM constants used for the $\mathrm{AA6061\mbox{-}T6}$ and $\mathrm {Al_2O_3}$ interfaces in this study are listed in Table 2.

**Table 2:** Parameters employed for CZM of matrix/particle interface.

Parameter	$K_{nn}$, $K_{ss}$, $K_{tt}$^39^	$T_n^0$^40^	$T_s^0$, $T_t^0$^40^	$G_c$^40^
Value	$2\times 10^7\; \mathrm {MPa\cdot {mm}^{-1}}$	4.57 $\textrm{GPa}$	2.93 $\textrm{GPa}$	15 $\mathrm {J/m^2}$

## Computational model

The $\textrm{FE}$ model and its validation are described in the following subsection. To investigate the damage mechanisms related to the failure modes of AA6061-T6/Al_2_O_3_ composites, a Python-generated script is used. This script enables the implementation of indentation-hardness test specifications to assess the resulting microstructure. Using this script, it is possible to generate particles with different distributions and volume fractions throughout the $\textrm{FGMMCs}$ structure.

**Figure 3:**
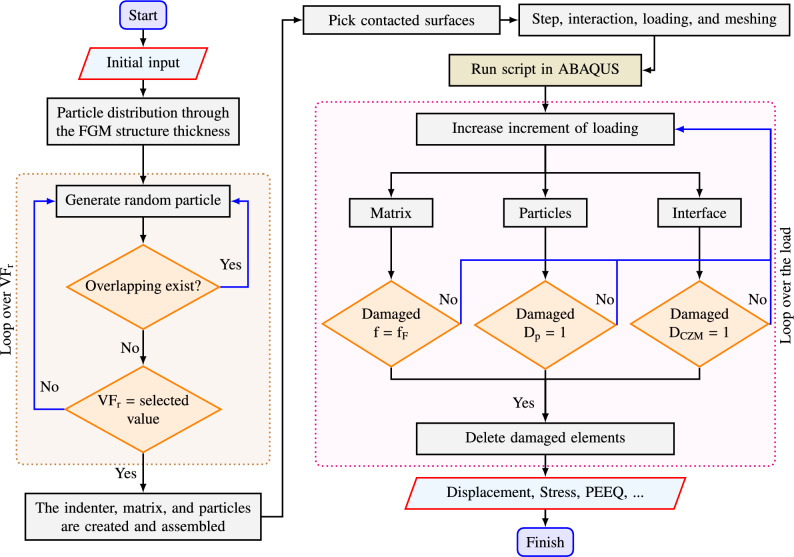
Flowchart of the present analysis.

Mathematical equations representing the different failure modes outlined in this study have been compiled and integrated into the ABAQUS $\textrm{FE}$ software. The analysis process is summarized in the flowchart presented in Fig. 3, providing an overview of the steps involved in the current study.

### Finite element model

**Figure 4:**
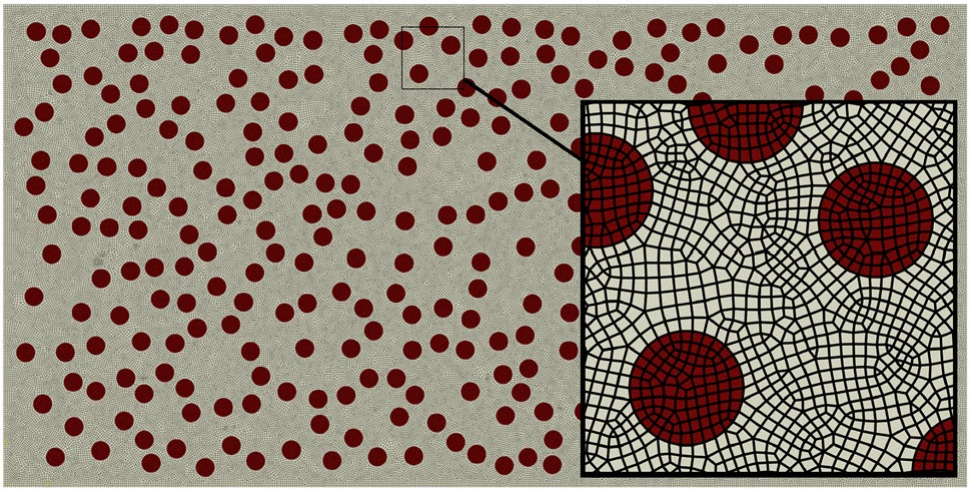
Finite element mesh for the studied model.

The analysis considered various factors such as geometric considerations, material nonlinearities, and nonlinear deformations caused by the contact between a rigid indenter and a metal matrix surface. The analysis employed a $\textrm{2D}$ $\textrm{FE}$ model with 4-node bilinear plane strain quadrilateral elements ($\textrm{CPE4R}$). To capture the damage behavior and failure modes precisely, a refined mesh with an element size of ${0.01}\,\hbox {mm}$ is employed throughout the microstructure. This resulted in an average of 146,700 elements and 156,000 nodes per microstructure (Fig. 4). In accordance with the findings of a previous investigation^41^, the optimal particle shape utilized in this study is circular with a diameter (d) of ${100}\,{\upmu }\hbox {m}$

**Figure 5:**
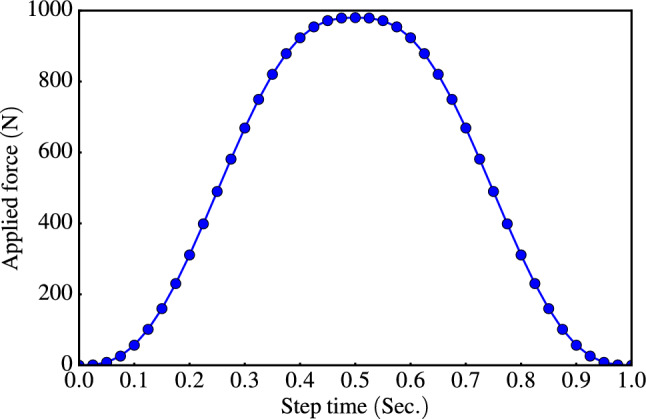
Applied force as a smooth step in ABAQUS.

To optimize computational efficiency, the $\textrm{FE}$ model employs a perfectly rigid indenter, and a fixed lower boundary is chosen for the structure to maintain stability. A Coulomb friction coefficient of 0.1^42,43^ is applied at both the indenter-matrix and particle-matrix interfaces to model frictional interactions. For quasi-static analysis in the ABAQUS software, the force was gradually applied in a smooth step^44^, as depicted in Fig. 5.

### Validation of the model

The accuracy of the $\textrm{FE}$ model is validated by comparing its predicted $\textrm{HRB}$ values with those obtained experimentally for the corresponding $\textrm{PRMMCs}$ reported by^45^. The measurement procedure defined in ASTM E18-22^24^ is employed in the experimental study. This method initially involves applying a minor load to establish a reference position, followed by the application of a major load for a specified time interval. Subsequently, the major load is removed and the difference in the penetration depth of the indenter is measured and used to calculate the $\textrm{HRB}$ value.

**Figure 6:**
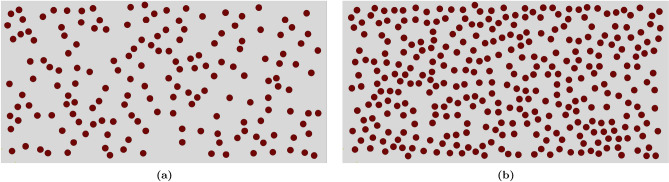
Representation of microstructure of $\mathrm{AA6061\mbox{-}T6/Al_2O_3}$ composite with various $\mathrm {VF_{r}}$.

For both volume fractions of 10% and 20%, at least *ten* random base models were considered in the experimental study, as the $\mathrm {Al_2O_3}$ particles were randomly dispersed within the structure (refer to Fig. 6). The average $\textrm{HRB}$ value was used for the comparison.

Table 3 presents a comparison between the random distribution of the proposed model and experimental data. The results indicate that the error ranges from 0.635 to 1.811% when the present random distribution is compared with the experimental data. This low percentage error indicates that the $\textrm{HRB}$ values obtained from the $\textrm{FE}$ model for volume fractions of 0%, 10%, and 20% are very close to the corresponding experimental values. The comparison demonstrates good agreement between the predicted and experimental results. This minimal error percentage error suggests that the $\textrm{HRB}$ values derived from the $\textrm{FE}$ model for volume fractions of 0%, 10%, and 20% closely align with the corresponding experimental values. The comparison demonstrates strong agreement between the predicted and experimental results. For further details about the model and its validation, please refer to the previous work^41^.

**Table 3:** HRB validation results.

$\mathrm {Al_2O_3\;VF_r}$	Experimental^45^	Present model random distribution	Error %
0%	$61\pm 0.4$	61.387	0.635
10%	$69\pm 0.2$	68.601	0.578
20%	$75\pm 0.9$	73.642	1.811

## Results and discussions

The main objective of this section is to explore the effect of $\mathrm {Al_2O_3}$ particle distribution on the mechanical characteristics and damage mechanisms of the $\mathrm{AA6061\mbox{-}T6/Al_2O_3}$ composite. To achieve this objective, this study analyzes the effects of the $\mathrm {Al_2O_3}$ particle distribution across the $\textrm{FGMMCs}$ structure on the mechanical properties of the composite.

An analysis is conducted to investigate the indentation behavior of structures composed of $\textrm{FGMMCs}$ under the influence of a spherical indenter. The structure under examination is modelled to exhibit a variation in the $\mathrm {VF_r}$ of the $\mathrm {Al_2O_3}$ particles throughout its thickness, as depicted in Fig. 2.

$\textrm{FGMMCs}$ are modelled using the linear rule of mixtures, which is a commonly employed approach^17^. This modelling technique assumes a linear relationship between the volume fractions of the $\mathrm {Al_2O_3}$ reinforcement and $\mathrm{AA6061\mbox{-}T6}$ matrix material. The sum of the volume fractions of the reinforcement and matrix material is equal to one and is expressed as

24 $$\begin{aligned} V_{rein}+V_{mat}=1, \end{aligned}$$

$\mathrm {V_{rein}}$ and $\mathrm {V_{mat}}$ represent the volume fractions of $\mathrm {Al_2O_3}$ and $\mathrm{AA6061\mbox{-}T6}$ matrix materials, respectively. In the investigated $\textrm{FGMMCs}$ structures (Fig. 2), the $\mathrm {Al_2O_3}$ particles are distributed randomly throughout the matrix. This irregularity in the arrangement of the reinforcement particles reflects the actual conditions observed in the $\textrm{FGMMCs}$.

The investigation focused on studying the variation in $\mathrm {VF_r}$ of $\mathrm {Al_2O_3}$ particles across the thickness of $\textrm{FGMMCs}$ using two approaches. The first approach is the power law approach^46^, whereas the second approach utilized a Gaussian distribution^47^.

The first approach (power law) is commonly employed in the theoretical modeling of $\textrm{FGMMCs}$ structures^17,48,49^. In this approach, the $\mathrm {VF_r}$ of $\mathrm {Al_2O_3}$ particles at any position $\textrm{y}$ across the thickness is assumed to be determined by the equation

25 $$\begin{aligned} VF_r(y)=0.3\times \left( \frac{y}{H}\right) ^{n}. \end{aligned}$$

Here, $\textrm{n}$ represents the non-negative compositional gradient exponent, which determines the variation profile of the $\mathrm {Al_2O_3}$ particles. Different values of $\textrm{n}$ result in distinct particle distribution profiles and overall particle volume fractions. Position $\textrm{y}$ indicates the location within the thickness of the $\textrm{FGMMCs}$ structure measured from its bottom surface. $\textrm{H}$ represents the total thickness of the $\textrm{FGMMCs}$ structure.

**Figure 7:**
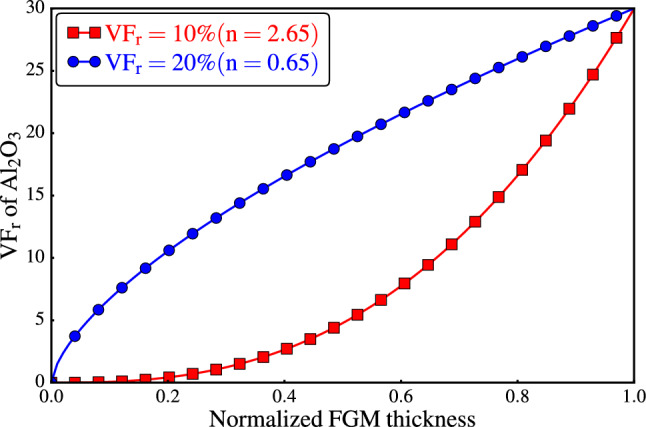
Compositional profiles of the FGMMCs using power law.

To enable meaningful comparisons between $\textrm{PRMMCs}$ and $\textrm{FGMMCs}$, two total particle volume fractions are considered, 10% and 20%. To determine the volume fractions, the compositional gradient exponent $\textrm{n}$ is estimated to be 2.65 for a total $\mathrm {VF_r}$ of 10%, whereas a value of 0.65 is estimated for $\textrm{n}$ when the total $\mathrm {VF_r}$ is 20%. Figure 7 shows the distribution curves obtained using the estimated values.

**Figure 8:**
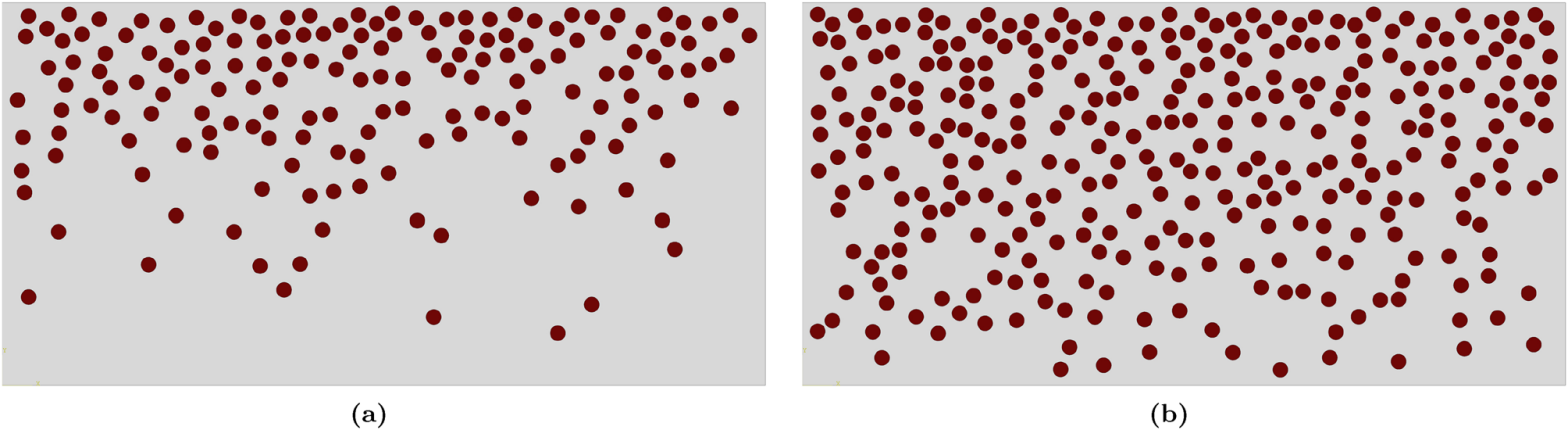
The distribution of $\mathrm {Al_2O_3}$ particles across the FGMMCs structure thickness using power law.

Figure 8 shows the samples representing $\textrm{FGMMCs}$ resulting from the application of the power law approach. A sample with a total $\mathrm {VF_r}$ of 10% ($\textrm{n}$ $=2.65$) is depicted in Fig. 8a, whereas a sample with a total $\mathrm {VF_r}$ of 20% ($\textrm{n}$ $=0.65$) is illustrated in Fig. 8b.

The second approach (Gaussian distribution) used to vary the $\mathrm {Al_2O_3}$ particle distribution within the thickness of the $\textrm{FGMMCs}$ structure is based on experimental work, specifically the study conducted by^11–13,16^ (see Fig. 1). In this approach, the $\mathrm {VF_r}$ distribution at any position $\textrm{y}$ is determined by fitting the experimental distribution curves. The best fit is achieved using a Gaussian distribution function^47^, which is represented by the following formula:

26 $$\begin{aligned} VF_r(y)=A_0\exp \left[ -\frac{(y-x_0)^2}{2\sigma _G^2}\right] . \end{aligned}$$

**Figure 9:**
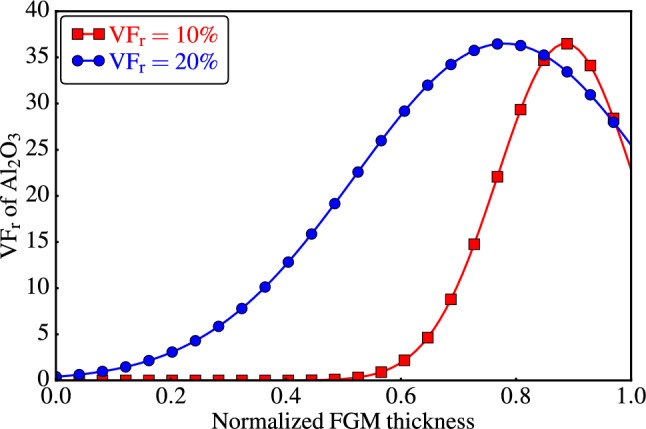
Compositional profiles of the FGMMCs using Gaussian distribution.

The constants $\mathrm {A_0}$, $\mathrm {x_0}$, and $\mathrm {\sigma _G}$ determine the shape of the Gaussian curve, and consequently, the particle volume fraction. These constants are estimated using trial methods to obtain specific volume fractions, these constants are estimated through trial methods. For a total $\mathrm {VF_r}$ of 10%, the estimated values of $\mathrm {A_0}$, $\mathrm {x_0}$, and $\mathrm {\sigma _G}$ are 0.365, 2.215, and 0.295, respectively. Similarly, for a total $\mathrm {VF_r}$ of 20%, the constants are estimated to be $\mathrm {A_0}$ $=0.365$, $\mathrm {x_0}$ $=1.95$, and $\mathrm {\sigma _G}$ $=0.65$. The distribution curves for $\mathrm {VF_r}$ values of both 10% and 20% using a Gaussian distribution are shown in Fig. 9.

**Figure 10:**
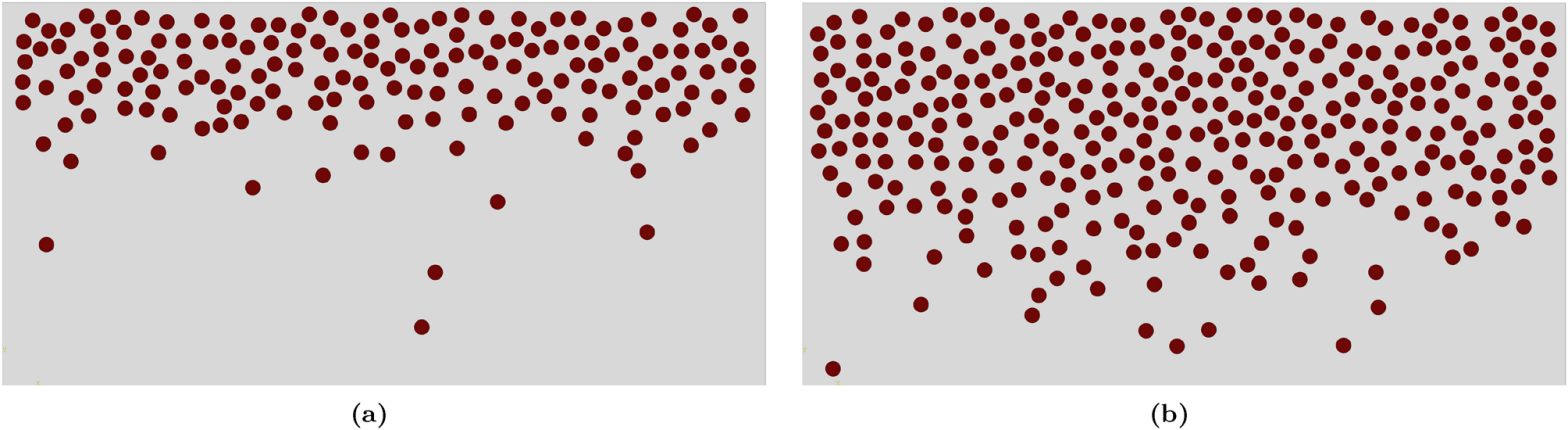
The distribution of $\mathrm {Al_2O_3}$ particles across the FGMMCs structure thickness using Gaussian distribution.

Representative samples of $\textrm{FGMMCs}$ structure with these $\mathrm {VF_r}$ variations obtained using the Gaussian approach are shown in Fig. 10. Figure 10a depicts a structure with a total $\mathrm {VF_r}$ of 10%, whereas Fig. 10b shows a structure with a total $\mathrm {VF_r}$ of 20%.

### HRB results

Nonlinear $\textrm{FE}$ analyses are performed to explore the influence of the power law and Gaussian distributions in $\textrm{FGMMCs}$ on the indentation behavior of $\mathrm{AA6061\mbox{-}T6/Al_2O_3}$ $\textrm{FGMMCs}$. Specifically, two total volume fractions of $\mathrm {Al_2O_3}$ particles are considered: 10% and 20%. This is achieved by varying the compositional gradient exponents ($\textrm{n}$ $\mathrm {= 0.65~and~2.65}$) for the power law distribution and by adjusting three parameters ($\mathrm {A_0}$, $\mathrm {x_0}$, and $\mathrm {\sigma _G}$) for the Gaussian distribution.

**Figure 11:**
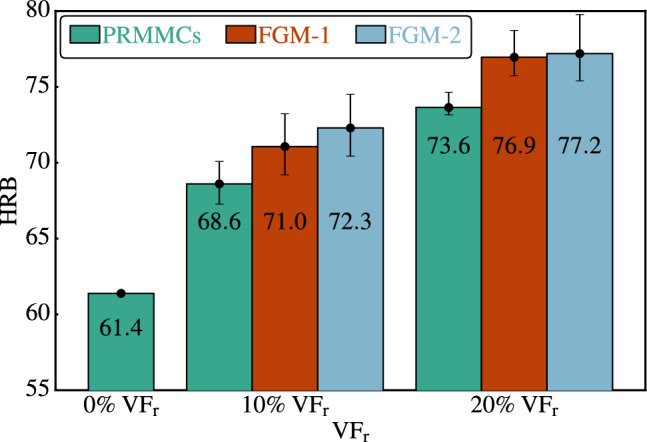
The variations of HRB for different $\mathrm {Al_2O_3}$ particles $\mathrm {VF_r}$ and variation through the FGMMCs thickness.

Ten samples with random reinforcement distributions are analyzed for each $\mathrm {VF_r}$ and distribution, and the calculated indentation depths are used to determine the $\textrm{HRB}$ values. Figure 11 presents the $\textrm{HRB}$ results for the pure $\mathrm{AA6061\mbox{-}T6}$ matrix as a reference value and the average $\textrm{HRB}$ values for the $\mathrm{AA6061\mbox{-}T6/Al_2O_3}$ composites with volume fractions of 10% and 20% $\mathrm {Al_2O_3}$ particles. In addition to the $\textrm{HRB}$ of the pure matrix (0% $\mathrm {VF_r}$), the $\textrm{HRB}$ results are shown three times in Fig. 11 for both the 10% and 20% particle $\mathrm {VF_r}$. The first set of results corresponds to the random distribution of $\mathrm {Al_2O_3}$ particles throughout the thickness without any variation in particle concentration, labeled as $\textrm{PRMMCs}$. The other two sets of $\textrm{HRB}$ results show the effects of particle variation through the thickness using the power law and Gaussian distribution, labeled FGM-1 and FGM-2, respectively.

According to the results presented in Fig. 11, the $\textrm{HRB}$ results exhibited a consistent pattern. Specifically, when examining volume fractions of 10% and 20%, it is apparent that the $\textrm{HRB}$ values of $\textrm{PRMMCs}$ are greater than those of the pure $\mathrm{AA6061\mbox{-}T6}$ matrix (0% $\mathrm {VF_r}$). Additionally, it can be observed that the $\textrm{HRB}$ values of the $\textrm{FGMMCs}$ using the power law distribution (labeled FGM-1) are higher than those achieved with the $\textrm{PRMMCs}$, and the $\textrm{HRB}$ values of $\textrm{FGMMCs}$ obtained through the Gaussian distribution (labeled FGM-2) are higher than those obtained through the power law distribution.

Table 4 shows a comparison between the $\textrm{HRB}$ results and their enhancement over the pure $\mathrm{AA6061\mbox{-}T6}$ matrix for all $\mathrm {Al_2O_3}$ particle volume fractions and variations through the structure thickness. It is shown that the $\textrm{HRB}$ is enhanced for a 10% $\mathrm {VF_r}$ over the pure matrix by 11.7%, 15.6%, and 17.8% for $\textrm{PRMMCs}$, FGM-1, and FGM-2, respectively. For 20% $\mathrm {VF_r}$, the enhancements are 19.8%, 25.2, and 25.7 for $\textrm{PRMMCs}$, FGM-1, and FGM-2, respectively. It is noted from the results in Table 4 that the hardness values in the case of the Gaussian distribution are greater than in the case of the power law distribution as a result of the convergence of the particles in the part closest to the surface of the samples, which leads to an increase in the resistance of the samples to penetration due to the increase in the hardest material in the upper part of the samples, this difference in results appears as a result of the difference in the distribution according to the shape of the functions, which is clearly shown in Figs. 7 and 9, as well as the distribution in samples representing the distributions in Figs. 8 and 10.

The results highlight the importance of $\textrm{FGMMCs}$, because the $\textrm{HRB}$ values obtained for $\textrm{FGMMCs}$ with a Gaussian distribution and a particle $\mathrm {VF_r}$ of 10% are comparable to the $\textrm{HRB}$ values obtained for $\textrm{PRMMCs}$ with a particle $\mathrm {VF_r}$ of 20%. The difference between the $\textrm{HRB}$ results is approximately 2%. This means that with a low particle $\mathrm {VF_r}$ and changing the particle variation across the material thickness, that is, by a Gaussian distribution, $\textrm{HRB}$ can be enhanced significantly.

As particle $\mathrm {VF_r}$ increases, it becomes evident that the $\textrm{HRB}$ values also increase. Notably, the results obtained for both distributions of $\textrm{FGMMCs}$ with a particle $\mathrm {VF_r}$ of 20% exhibited minimal differences. This observation indicates that it is challenging to achieve a significant difference in the $\textrm{HRB}$ values through particle distribution for $\textrm{FGMMCs}$ with higher particle volume fractions such as 20%.

**Table 4:** HRB results for various particles $\mathrm {VF_r}$ and variation across the MMCs thickness.

$\mathrm {VF_r}$	HRBPRMMCs	Enhancement % over pure matrix	HRB FGMMCs Power law	Enhancement % over pure matrix	HRB FGMMCs Gaussian	Enhancement % over pure matrix
10%	68.6	11.7	71.0	15.6	72.3	17.8
20%	73.6	19.8	76.9	25.2	77.2	25.7

**Figure 12:**
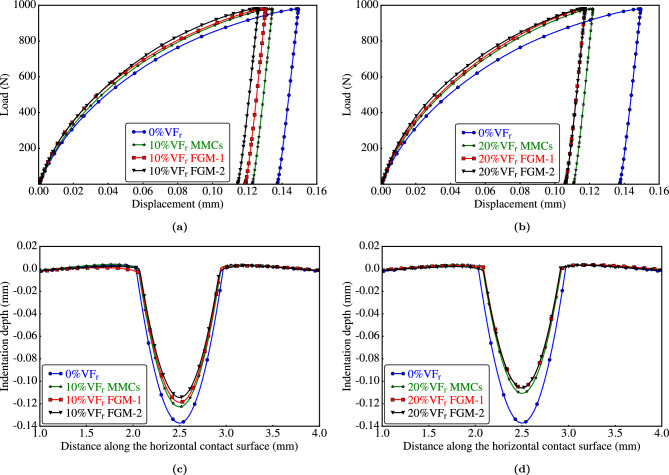
Effects of $\mathrm {VF_r}$ of $\mathrm {Al_2O_3}$ particles on load-displacement curves and deformed indentation surfaces.

The load-displacement and surface profiles curves of representative specimens are shown in Fig. 12. The indentation depth decreased significantly as $\mathrm {VF_r}$ increased. Notably, distinct variations in the results are evident for all representative samples with a $\mathrm {VF_r}$ of 10%, as illustrated in Fig. 12a,c. Conversely, for a $\mathrm {VF_r}$ of 20%, the curves for the two representative samples of $\textrm{FGMMCs}$ are nearly identical, as depicted in Fig. 12b,d.

### Residual von-Mises stress and plastic strain

**Figure 13:**
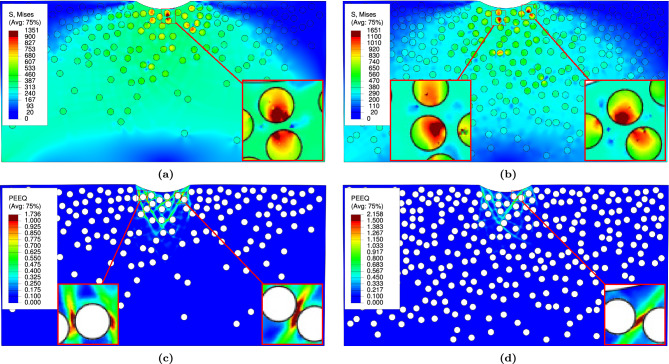
Effect of power law distribution on residual von-Mises stress and effective plastic strain during loading.

Figure 13 illustrates the effect of $\mathrm {VF_r}$ on the distributions of residual von-Mises stress and effective plastic strain within the $\textrm{FGMMCs}$ structure. As $\mathrm {VF_r}$ decreases, the levels of residual stress and strain in the representative samples decrease. It is worth noting that the regions of the effective plastic strain are localized beneath the indenter and propagate at an angle of nearly ${45}^{\circ }$.

The stress concentration is influenced by the relative distances between the particles and indenter as well as between the particles themselves. Consequently, as $\mathrm {VF_r}$ increases, $\textrm{FGMMCs}$ becomes more susceptible to stress concentration. This is because the permissible area for particle movement is reduced with higher particle $\mathrm {VF_r}$. Moreover, the particles located beneath the indenter experienced the most significant effects, as shown in Fig. 13b,d.

The range of von-Mises stress varies from 1010 $\hbox {MPa}$ to 1565 $\hbox {MPa}$ for a particle $\mathrm {VF_r}$ of 10% ($\textrm{n}$ $=2.65$), and for a particle $\mathrm {VF_r}$ of 20% ($\textrm{n}$ $=0.65$), with a range of 1282 $\hbox {MPa}$ to 3215 $\hbox {MPa}$, as shown in Table 5. The effective plastic strain ranges from 1.566 to 2.41 for a $\mathrm {VF_r}$ of 10% ($\textrm{n}$ $=2.65$), and 1.507 to 2.524 for $\mathrm {VF_r}$ of 20% ($\textrm{n}$ $=0.65$) (see Table 5).

**Figure 14:**
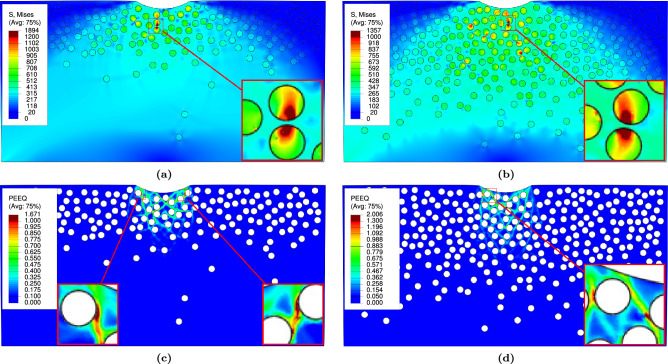
Effect of Gaussian distribution on residual von-Mises stress and effective plastic strain during loading.

Figure 14 presents the effects of the Gaussian distribution on the distributions of the residual von-Mises stress and effective plastic strain within the $\textrm{FGMMCs}$ structure for both 10% and 20% volume fractions. Similar to previous observations, localized stress concentration areas beneath the indenter and the propagation angle of the effective plastic strain are evident in these results (see Fig. 14b,d).

By analyzing the distribution of von-Mises stress for the representative samples (Fig. 14a,b), it is observed that the stress levels are higher for 10% $\mathrm {VF_r}$. This can be attributed to the chosen Gaussian distribution, which results in a higher concentration of particles in the region directly below the indenter compared with the distribution with a 20% $\mathrm {VF_r}$. This can be observed by noting the positions of the peak values in the curves, as depicted in Fig. 9.

The range of von-Mises stress for $\textrm{FGMMCs}$ with a Gaussian distribution is 1401 $\hbox {MPa}$ to 2446 $\hbox {MPa}$ for a particle $\mathrm {VF_r}$ of 10% and 1196 $\hbox {MPa}$ to 2228 $\hbox {MPa}$ for a particle $\mathrm {VF_r}$ of 20%, as shown in Table 6. The effective plastic strain ranged from 1.615 to 2.865 for the 10% particle $\mathrm {VF_r}$ and 1.331 to 2.474 for the 20% particle $\mathrm {VF_r}$ (see Table 6). These values indicate the extent of plastic deformation experienced by the matrix material in the $\textrm{FGMMCs}$ structures with Gaussian distributions.

A comparison of the von-Mises stress results between $\textrm{FGMMCs}$ and $\textrm{PRMMCs}$ showed that there is a significant increase in the von-Mises stress values in the case of $\textrm{FGMMCs}$. For instance, when comparing the average von-Mises stress values in both cases, it is found that with 10% $\mathrm {VF_r}$, the von-Mises stress values increased by 64% and 117% for $\textrm{FGMMCs}$ with power law and Gaussian distributions, respectively. Similarly, with 20% $\mathrm {VF_r}$, the average value of the von-Mises stress increased by 81% and 57% for both the power law and Gaussian distributions, respectively.

Furthermore, an increase in the average values of effective plastic strain is also observed. When considering a 10% $\mathrm {VF_r}$ of particles, the effective plastic strain increased by 84% and 90% for the power law and Gaussian distribution, respectively. Similarly, with 20% $\mathrm {VF_r}$, the effective plastic strain for power law and Gaussian distribution increased by 35% and 37%, which indicates the tendency to damage for $\textrm{FGMMCs}$ over $\textrm{PRMMCs}$.

### Damage analysis of FGMMCs

This subsection presents an analysis of the existing and potential damage locations within the AA6061-T6/$\mathrm {Al_2O_3}$ $\textrm{FGMMCs}$ structure for three potential failure modes: (i) matrix damage, (ii) particle fracture, and (iii) interfacial decohesion. The results of the damage parameters associated with each failure mode are analyzed.

**Figure 15:**
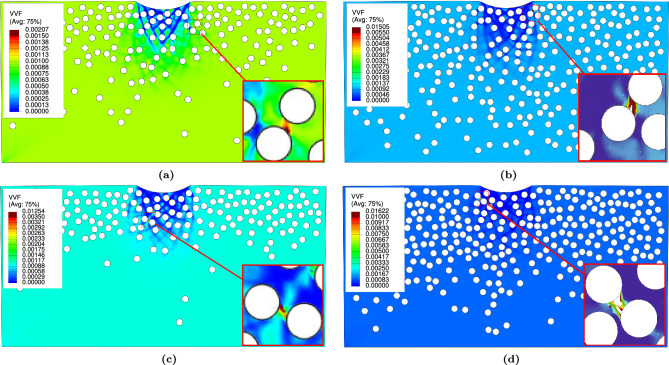
Void volume fraction in $\mathrm {AA6061-T6}$ matrix for power law and Gaussian during loading.

(i) Matrix damage:

For $\mathrm {VF_r}$ of 10%, no damage is observed in any sample for both $\textrm{FGMMCs}$ distribution approaches. Fig. 15a,c shows the representative samples for this $\mathrm {VF_r}$, which help predict the failure mechanisms in the $\mathrm{AA6061\mbox{-}T6}$ matrix. As shown in Table 5, the range of the void volume fraction ($\textrm{VVF}$) for a $\mathrm {VF_r}$ of 10% ($\textrm{n}$ =2.65) is between 0.00121 and 0.00398, with an average value of 0.00216. Considering the results of $\textrm{VVF}$ for the Gaussian $\textrm{FGMMCs}$ distribution, the range of $\textrm{VVF}$ is between 0.00115 and 0.01254, with an average value of 0.00299 for a $\mathrm {VF_r}$ of 10% (see Table 6).

**Figure 16:**
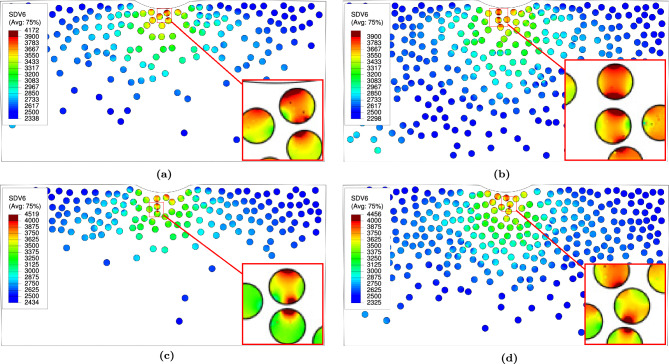
Yield stress distribution in $\mathrm {Al_2O_3}$ particles for power law and Gaussian during loading.

Based on the results, it is observed that the matrix damage mode occurred when $\mathrm {VF_r}$ is 20% for both the power law and Gaussian distributions of the $\textrm{FGMMCs}$. Out of the ten samples studied for each distribution, only one sample in each case exhibited clear damage. These two samples ($\mathrm {S_2~and~S_7}$) are shown in Fig. 15b,d. For a $\mathrm {VF_r}$ of 20% ($\textrm{n}$ =0.65), the range of the $\textrm{VVF}$ is between 0.00229 and 0.06, with an average value of 0.012353 (Table 5). These findings indicated that a higher $\mathrm {VF_r}$ leads to a higher possibility of void nucleation and growth. For a $\mathrm {VF_r}$ of 20% Gaussian $\textrm{FGMMCs}$ distribution, the range is between 0.00162 and 0.06, with an average value of 0.00913 (see Table 6). The higher possibility of matrix damage in these cases is attributed to the increase in the number of particles, which reduces the distance between them owing to the limited space available for particle movement.

(ii) Particle fracture:

The $\textrm{JH2}$ damage parameter for the particles is found to be zero for both volume fractions (10% and 20%) in the two types of $\textrm{FGMMCs}$ with the power law and Gaussian distributions. The distribution of the $\textrm{JH2}$ yield stress parameter for the particles is shown in Fig. 16, which indicates critically loaded particles that are susceptible to damage.

For samples with a power law distribution (represented in Fig. 16a,b), the range of the yield stress parameter is between 3958 $\hbox {MPa}$ and 4971 $\hbox {MPa}$, with an average value of 4309 $\hbox {MPa}$ for a $\mathrm {VF_r}$ of 10% ($\textrm{n}$ $= 2.65$), as shown in Table 5. For a $\mathrm {VF_r}$ of 20% ($\textrm{n}$ $= 0.65$), the range is between 3994 $\hbox {MPa}$ and 5207 $\hbox {MPa}$, with an average value of 4488 $\hbox {MPa}$ (see Table 5).

**Figure 17:**
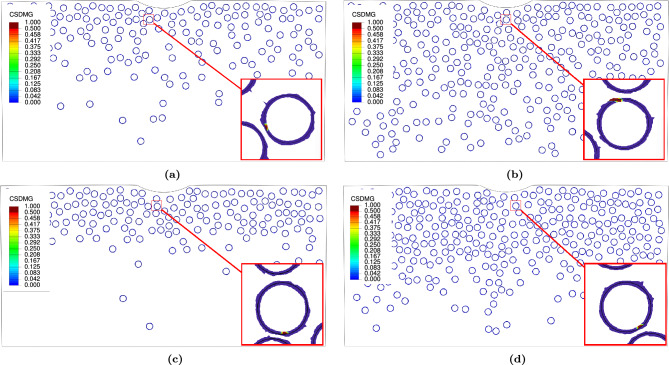
Spatial distribution of matrix/particle decohesion damage parameter (CSDMG) for FGMMCs during Loading.

For samples with a Gaussian distribution (represented in Fig. 16c,d), the range of the yield stress parameter is between 4104 $\hbox {MPa}$ and 5651 $\hbox {MPa}$, with an average value of 4530 $\hbox {MPa}$ for a $\mathrm {VF_r}$ of 10% (see Table 6). For a $\mathrm {VF_r}$ of 20%, the range is between 4040 $\hbox {MPa}$ and 4993 $\hbox {MPa}$, with an average value of 4517 $\hbox {MPa}$. These findings provide insight into the distribution of the yield stress parameter, highlighting the potential susceptibility of critically loaded particles to damage in both the power law and Gaussian $\textrm{FGMMCs}$.

(iii) Interfacial decohesion:

For samples with a power law distribution and 10% $\mathrm {VF_r}$ ($\textrm{n}$ $= 2.65$), there are three samples ($\mathrm {S_2,~S_5,~and~S_6}$) in which complete interfacial decohesion is observed. One of these samples ($\mathrm {S_6}$) is shown in Fig. 17a. Two samples ($\mathrm {S_4~and~S_8}$) exhibited interfacial decohesion when the $\mathrm {VF_r}$ is 20% ($\textrm{n}$ $=0.65$), with one of the samples ($\mathrm {S_4}$) shown in Fig. 17b.

Regarding the Gaussian distribution, interfacial decohesion is observed in three samples ($\mathrm {S_3,~S_6,~and~S_7}$) with a $\mathrm {VF_r}$ of 10%, and one sample ($\mathrm {S_7}$) with a $\mathrm {VF_r}$ of 20$\%$. Figure 17c,d shows one sample for each $\mathrm {VF_r}$. These findings indicate that interfacial decohesion is a prevalent phenomenon in $\textrm{FGMMCs}$ regardless of the distribution type and $\mathrm {VF_r}$.

### Comparative analysis of the power law and Gaussian distribution

To compare the two distribution approaches for $\textrm{FGMMCs}$ (the power law and Gaussian distributions), several factors are considered. First, the $\textrm{HRB}$ values are examined, which indicated that the Gaussian distribution approach is the preferred method, particularly for a $\mathrm {VF_r}$ of 10%. Additionally, to obtain a fair understanding of the preferred distribution approach, the damage incurred by the three different modes in the $\textrm{FGMMCs}$ is studied. Because damage does not occur uniformly across all modes, a normalization procedure is conducted for each damage parameter^50^. For each $\mathrm {VF_r}$, the damage parameter, or the parameter indicating the occurrence of damage, is normalized based on the results obtained from the samples distributed according to both the power law and Gaussian approaches. The following normalization formula of^50^ is used

27 $$\begin{aligned} X_{norm} = \frac{X-X_{min}}{X_{max}-X_{min}}. \end{aligned}$$

**Figure 18:**
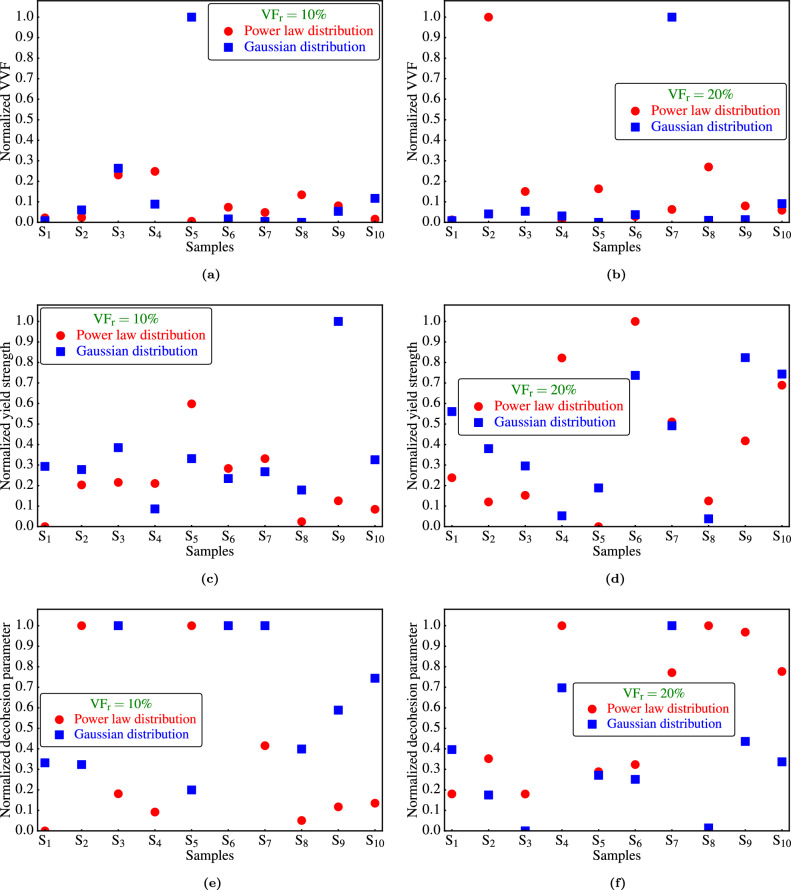
Comparison between the two approaches of FGMMCs distribution.

Here, $\mathrm {X_{norm}}$ represents the normalized value, $\textrm{X}$ denotes the set of values used for normalization, $\mathrm {X_{min}}$ represents the minimum values within $\textrm{X}$, and $\mathrm {X_{max}}$ represents the maximum values within $\textrm{X}$. By employing this normalization process, a fair comparison between the damage parameters obtained from the two distribution approaches is achieved, allowing for a complete evaluation of the preferred distribution approach for $\textrm{FGMMCs}$. Figure 18 presents the normalized values obtained for each distribution approach and $\mathrm {VF_r}$.

Based on the analysis of Fig. 18a,c,e for a $\mathrm {VF_r}$ of 10%, it can be concluded that $\textrm{FGMMCs}$ with a Gaussian distribution are more susceptible to damage, particularly interfacial decohesion (as depicted in Fig. 18e). This is because the higher susceptibility to damage in the Gaussian distribution of samples with a particle $\mathrm {VF_r}$ of 10% can be attributed to the fact that 60% of these samples exhibited more than 40% damage. In contrast, for samples following a power law distribution, the percentage of samples that experienced damage is lower (approximately 30%). Therefore, for a $\mathrm {VF_r}$ of 10%, the Gaussian distribution offers higher hardness, but is also more susceptible to damage, although it does not necessarily ensure the occurrence of such damage.

From the examination of Fig. 18b,d,f, it is evident that the Gaussian distribution exhibits lower susceptibility to damage. Hence, for a $\mathrm {VF_r}$ of 20%, a Gaussian distribution is a favorable choice to achieve both high hardness and improved resistance against damage. Considering these results, decisions regarding the distribution approach should consider the specific requirements of the application and evaluate the desired hardness against an acceptable level of potential damage.

**Table 5:** Output values for FGMMCs using power law distribution.

Samples	FGMMCs using Power law distribution									
	$\mathrm {10\%~VF_r~(n=2.65)}$					$\mathrm {20\%~VF_r~(n=0.65)}$				
	von−Mises	PEEQ	VVF	$\mathrm {Particle_{yield}}$	$\mathrm {CZM_{damage}}$	von−Mises	PEEQ	VVF	$\mathrm {Particle_{yield}}$	$\mathrm {CZM_{damage}}$
$S_1$	1010	1.838	0.0014	3958	0.25259	1485	1.7888	0.00232	4283	0.48826
$S_2$	1115	2.182	0.00142	4302	1	1386	1.565	0.06	4140	0.5955
$S_3$	1401	2.196	0.00378	4323	0.38791	1651	2.158	0.01041	4179	0.48805
$S_4$	1346	2.41	0.00398	4314	0.32125	2034	1.767	0.00229	4991	1
$S_5$	1400	1.566	0.00121	4971	1	1282	1.507	0.01117	3994	0.55584
$S_6$	1454	1.757	0.00199	4438	1	3215	2.089	0.0033	5207	0.57758
$S_7$	1565	1.756	0.0017	4519	0.56296	1746	2.514	0.0053	4613	0.85727
$S_8$	1131	1.867	0.00268	3999	0.29048	1475	2.524	0.01737	4146	1
$S_9$	1350	1.736	0.00207	4171	0.34019	1891	1.567	0.0063	4501	0.98032
$S_{10}$	1307	2.139	0.00133	4101	0.35345	1920	1.539	0.00507	4830	0.86073
Min.	1010	1.566	0.00121	3958	0.25259	1282	1.507	0.00229	3994	0.48805
Max.	1565	2.41	0.00398	4971	1	3215	2.524	0.06	5207	1
Avg.	1307.9	1.9447	0.00216	4309.6	0.551	1808.5	1.90188	0.013	4488.4	0.741

**Table 6:** Output values for FGMMCs using Gaussian distribution.

Samples	FGMMCs using Gaussian distribution									
	$\mathrm {10\%~VF_r~(n=2.65)}$					$\mathrm {20\%~VF_r~(n=0.65)}$				
	von−Mises	PEEQ	VVF	$\mathrm {Particle_{yield}}$	$\mathrm {CZM_{damage}}$	von−Mises	PEEQ	VVF	$\mathrm {Particle_{yield}}$	$\mathrm {CZM_{damage}}$
$S_1$	1645	1.735	0.00124	4455	0.5005	1547	1.775	0.00212	4674	0.62322
$S_2$	1765	1.96	0.00184	4429	0.49425	1356	2.006	0.004	4455	0.4851
$S_3$	1722	1.822	0.00415	4610	1	1411	2.277	0.00479	4353	0.37578
$S_4$	1413	1.917	0.00216	4104	0.64823	1343	2.266	0.00345	4058	0.8111
$S_5$	1894	1.671	0.01254	4519	0.4018	1464	1.331	0.00162	4223	0.54484
$S_6$	1401	2.863	0.00134	4355	1	1741	1.867	0.00381	4888	0.5326
$S_7$	1676	1.615	0.0012	4411	1	1547	1.752	0.06	4590	1
$S_8$	1489	1.648	0.00115	4260	0.55083	1196	1.76	0.00218	4040	0.38482
$S_9$	2446	1.927	0.00176	5651	0.69258	1811	1.739	0.00239	4993	0.64814
$S_{10}$	1841	2.865	0.00248	4510	0.80866	2228	2.474	0.00694	4896	0.58603
Min.	1401	1.615	0.00115	4104	0.4018	1196	1.331	0.00162	4040	0.37578
Max.	2446	2.865	0.01254	5651	1	2228	2.474	0.06	4993	1
Avg.	1729.2	2.0023	0.003	4530.4	0.71	1564.4	1.9247	0.01	4517	0.6

## Conclusion

A novel finite element model based on a random microstructure is proposed to investigate the deformation and damage behavior of functionally graded metal matrix composites subjected to indentation loading. This study focuses on the influence of the volume fraction and distribution percentage of $\mathrm {Al_2O_3}$ particles on the thickness of the composite structure. To the best of our knowledge, this study represents the first investigation into the influence of particle volume fraction and distribution on the damage mechanisms of $\mathrm{AA6061\mbox{-}T6/Al_2O_3}$ functionally graded metal matrix composites while considering various damage modes occurring during the indentation process. The model incorporated the Gurson-Tvergaard-Needleman (GTN) damage model to simulate the elastoplastic behavior and damage of the matrix, the JH2 cracking model to represent particle fracture, and the cohesive zone model (CZM) to account for matrix-particle interfacial decohesion. The model was employed in ABAQUS/Explicit software using a Python script. The significant findings of this study are as follows:

- The finite element model effectively captures the three primary damage mechanisms in $\textrm{PRMMCs}$, particularly in $\textrm{FGMMCs}$, thereby providing valuable insights into optimizing their performance.
- The hardness value ($\textrm{HRB}$) increases as the particle volume fraction and size increase. Additionally, the probability of fracture for the $\mathrm {Al_2O_3}$ particles increases with an increase in the volume fraction.
- $\mathrm {Al_2O_3}$ particles enhance the resistance of $\textrm{FGMMCs}$ to deformation, although their effectiveness is influenced by the particle volume fraction.
- $\textrm{PRMMCs}$ can be represented using a variation in the particle concentration throughout the thickness of the structure, resulting in $\textrm{FGMMCs}$. $\textrm{FGMMCs}$ exhibit a higher hardness value ($\textrm{HRB}$) than traditional Metal Matrix Composites ($\textrm{MMCs}$).
- This study demonstrates that with a volume fraction of 10% particles and a Gaussian distribution for particle variation through the structure thickness, the $\textrm{HRB}$ is approximately equivalent to the $\textrm{HRB}$ achieved with a volume fraction of 20% particles in $\textrm{MMCs}$ without any variation in particle distribution through the thickness.
- $\textrm{FGMMCs}$ with a Gaussian distribution yield a higher $\textrm{HRB}$ than those with a power-law distribution and exhibit closer alignment with the experimental distribution functions.

## Data Availability

The datasets used during the current study available from the corresponding author on reasonable request.
